# Stress and Microstructures Characterization Based on Magnetic Incremental Permeability and Magnetic Barkhausen Noise Techniques

**DOI:** 10.3390/ma17112657

**Published:** 2024-05-31

**Authors:** Hongwei Sheng, Ping Wang, Yuan Yang, Chenglong Tang

**Affiliations:** 1 College of Automation Engineering, Nanjing University of Aeronautics and Astronautics, Nanjing 210016, China; zjwx521@sina.com; 2 Key Laboratory of Non-Destructive Testing and Monitoring Technology of High-Speed Transportation Facilities of the Ministry of Industry and Information Technology, Nanjing 210016, China; 3 Central Research Institute of Baosteel, Shanghai 201999, China; tangcl@baosteel.com

**Keywords:** microstructure, stress, micro-magnetic non-destructive testing (NDT), magnetic domain feature, pattern recognition

## Abstract

Both microstructure and stress affect the structure and kinematic properties of magnetic domains. In fact, microstructural and stress variations often coexist. However, the coupling of microstructure and stress on magnetic domains is seldom considered in the evaluation of microstructural characteristics. In this investigation, Magnetic incremental permeability (MIP) and magnetic Barkhausen noise (MBN) techniques are used to study the coupling effect of characteristic microstructure and stress on the reversible and irreversible motions of magnetic domains, and the quantitative relationship between microstructure and magnetic domain characteristics is established. Considering the coupling effect of microstructure and stress on magnetic domains, a patterned characterization method of microstructure and stress is innovatively proposed. Pattern recognition based on the Multi-layer Perceptron (MLP) model is realized for microstructure and stress with an accuracy rate higher than 97%. The results show that the pattern recognition accuracy of magnetic domain features and micro-magnetic features simultaneously as input parameters is higher than that of micro-magnetic features alone as input parameters.

## 1. Introduction

Magnetic properties are sensitive to microstructure as well as stress [1,2,3]. However, in practical applications, changes in material microstructure are often accompanied by changes in stress. Thus, it is not simple to directly relate magnetic properties to stress and microstructure. Domain wall motion (reversible and irreversible) can be impeded by stress-induced pinning effects [4,5]. The accompanying “hardening” of the microstructure produces a high dislocation density, which also affects the motion of the magnetic domains. If the microstructure characteristics of the material are not known, the stress evaluation will lead to great misjudgment, leading to inaccurate stress predictions [1]. Neglecting the effect of stress on micro-magnetic nondestructive testing techniques, the accuracy of microstructure characterization will also be reduced.

The challenging task of stress and microstructure measurement is the properties characterization of the microscopic magnetic. The microstructure determines the micro-magnetic signal by determining the magnetic domain structure and motion properties (reversible and irreversible motions). Therefore, the characterization of magnetic domains with reversible and irreversible motion provides feedback on stress and microstructure information. P. Haušild argued that since both the stress in martensite and the volume fraction of martensite contribute to MBN signal, quantifying the residual stress in absolute way could only be possible if both the calibration curve of the MBN versus stress and the volume fraction of martensite were known [1]. L. Jia, based on MBN, investigated characters of the domain wall inside grains and around grain boundaries under low-tensile stress [5]. A. N. Stashkov regarded MIP as indicator of compression stress in low-carbon steel [6]. Benjamin Ducharne characterized the stress changes in iron–cobalt magnetic sheet based on MIP [7]. Tetsuya Uchimoto evaluated the creep-induced microstructural changes in 12Cr-Mo-V-W microstructure under different creep test conditions by using the MIP method [8,9]. G. Dobmann et al. [10,11,12,13,14] detected the incremental permeability of the material based on hysteresis loops using eddy current coils and proposed a semi-analytical modeling method, and fused the MIP with MBN and other detection techniques to create the 3MA detection method. MIP and MBN can be used to characterize the stress and microstructure.

However, very few studies in the current research have considered different microstructures and stress states simultaneously. In this paper, the coupling effect of different microstructures and stresses is analyzed based on two detection techniques, MIP and MBN. However, in practical applications, microstructure changes are accompanied by stress changes, and it is difficult to separate microstructure and stress. In this paper, by manually labeling different microstructures and different stress states with pattern recognition based on MLP model, simultaneous evaluation of material microstructure and stress states is achieved.

The rest of this paper is organized as follows: the experimental scheme, detection system, and MLP model are described in detail in Section 2. In Section 3, the NDT results and magnetic domain observations are analyzed and studied. The conclusions of the study are presented in Section 4.

## 2. Methods

### 2.1. Samples

**Figure 1:**
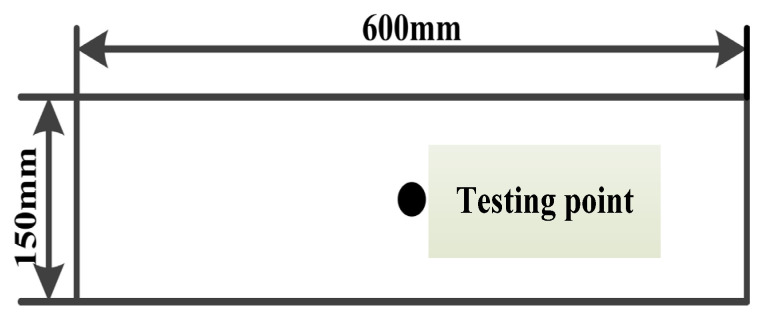
Sample size.

Four different cold rolled automotive sheet steels were prepared and cut to 150 × 600 mm^2^ size (Figure 1). To eliminate the residual stress, the samples were annealed in an annealing furnace at 500–600 ℃ for 4–5 h. The mechanical properties of samples are shown in Table 1. Sample 1 is ferrite steel, sample 2 is ferrite and pearlite steel, and samples 3 and 4 are martensite and ferrite steel. Due to the different microstructure, the mechanical properties of the samples are quite different.

### 2.2. MBN and MIP Testing

Ferromagnetic materials (composed of different phases) have a crystal-like structure. Ferrite phase structure is a body-centered cubic structure, while cementite is a complex orthogonal lattice. The crystal structure of lamellar pearlite is a complex rhombic structure. Between adjacent atoms, metamagnetic moments are generated due to electron spin, and there is an interaction force between the metamagnetic moments, which drives the adjacent metamagnetic moments to align in the same direction in parallel, forming magnetic domains. In the absence of an external magnetic field, the domains are balanced with each other and the magnetization intensity is equal to zero. The magnetization of the external magnetic field causes the displacement of the domain walls and the overturning of the domains to reach the magnetic saturation state. With different phase electron coordination number, the magnetic domain and magnetic domain interaction force are also different. Therefore, the motion states of magnetic domains under magnetic field excitation are different for different phases.

**Figure 2:**
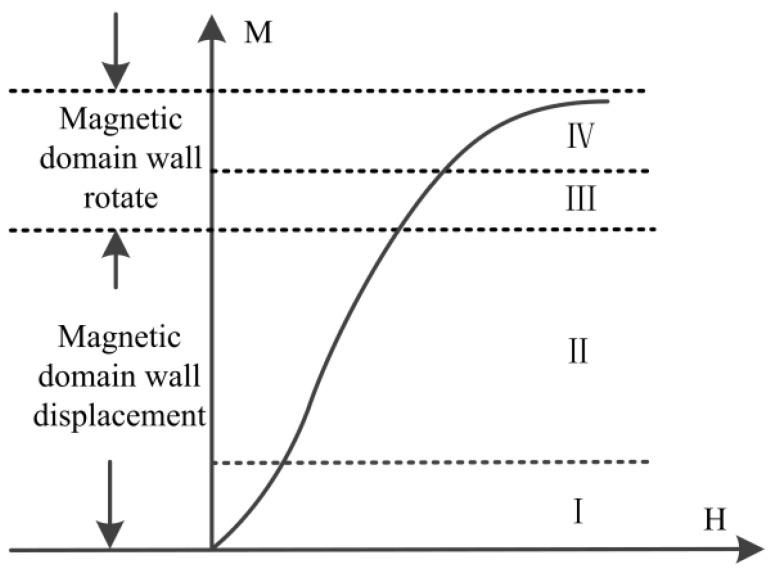
Magnetization of ferromagnetic materials.

The whole magnetization process can be described by the magnetization curve shown in Figure 2, including reversible domain wall displacement (I), irreversible domain wall displacement (II), reversible domain rotation (III), and near saturation (IV). The domain motion in the four stages (or the entire magnetization curve) is affected not only by the microstructure, but also by the stress state.

Under the action of excitation magnetic field, the signals of micro-magnetic NDT are different due to the different movement of magnetic domains, which is the basis for the characterization of the microstructure and stress state of ferromagnetic materials by the micro-magnetic NDT signals [15,16]. The whole magnetization process (Figure 2) involves both reversible and irreversible movements of magnetic domains. The single-principle testing technology can only reflect the motion characteristics of one magnetic domain in the whole magnetization period, for example, MBN technology can only extract the irreversible motion characteristics of magnetic domain. Therefore, with the use of single-principle testing technology, there are certain limitations. The extraction and analysis of micro-magnetic features that characterize the reversible and irreversible motion of magnetic domains, based on multi-source micro-magnetic NDT, can improve the accuracy of assessing the microstructure and stress state of ferromagnetic materials. The MBN signal, generated by the irreversible motion of the magnetic domain walls, could be used to describe the microstructure and the stress state of the ferromagnetic material [17,18,19,20,21,22,23,24]. Correspondingly, the MIP signal, generated by the reversible motion of the domain walls, could be also used to describe the microstructure and the stress state of the ferromagnetic material [25,26,27,28,29,30,31,32].

**Figure 3:**
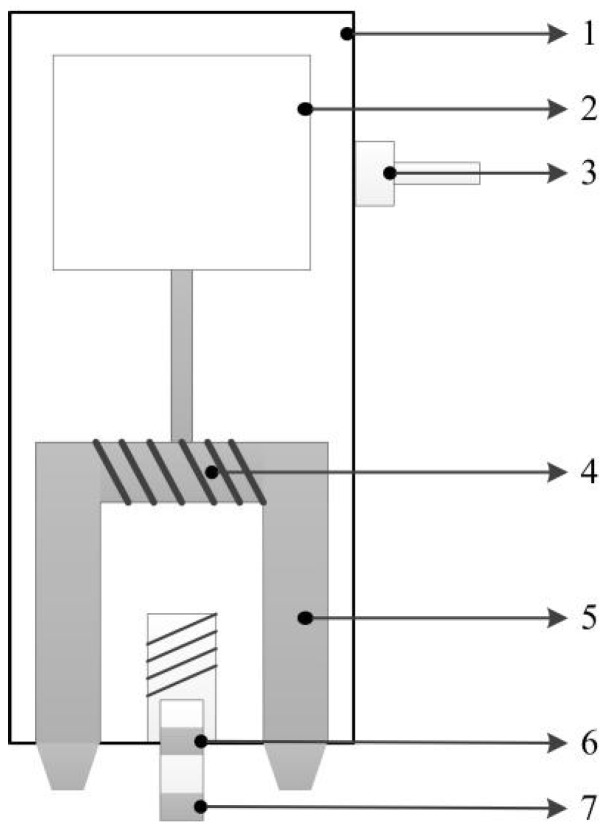
The probe of 3MA (simplified). 1—Housing, 2—Electronic board (preamp), 3—Connection cable, 4—Micromagnetic coils, 5—Magnetic yoke, 6—Transmitter coils, and 7—Receiver coil.

3MA was used for the NDT experiments to extract MBN and MIP signals [10,11,12,13,14]. The excitation coil (4, Figure 3) is wound on a U-shaped yoke (5, Figure 3). High-amplitude low-frequency (10–1000 Hz) sine excitation is applied to the excitation coil to generate a magnetic field sufficient to cause irreversible motion of the magnetic domains. The signal generated by the irreversible motion of the magnetic domains is picked up by the receiver coil (7, Figure 3). The MBN signals are processed using band-pass filters, a combination of low-pass/high-pass filters and amplified, which also are rectified including post-amplification and signal smoothing.

Unlike MBN, high- and low-frequency sinusoidal currents are necessary in MIP technology to obtain reversible domain motion information. A high-amplitude low-frequency (10–1000 Hz) excitation is applied to the excitation coil (4, Figure 3) generating a magnetic hysteresis loop in the material, as in case of the MBN method. Simultaneously, a sinusoidal current with low amplitude (mA levels), and high frequency (10 kHz−1 MHz) is fed into transmitter coils (6, Figure 3), which produces minor asymmetric hysteresis loops that superimpose to the major hysteresis curve [1]. The signal generated by the reversible motion of the magnetic domains is picked up by the receiver coil (7, Figure 3).

The butterfly curve [10] can be plotted with the excitation magnetic field intensity as the abscissa and the amplitude of the MBN or MIP signal as the ordinate coordinate during a magnetization cycle. The maximum amplitude (MMAX or UMAX) is derived as one test statistic. Correspondingly, the magnetic field strength at MMAX (UMAX) is assigned to the test statistic HCM (HCU). The expansions of the profile curve are evaluated at 25%, 50%, and 75% of MMAX (UMAX). The amplitude of the signal when the excitation field strength is 0 is called the remanent magnetism (MR or UR). An additional test statistic is MMEAN (UMEAN), which is the average value of the profile curve over a given period.

**Figure 4:**
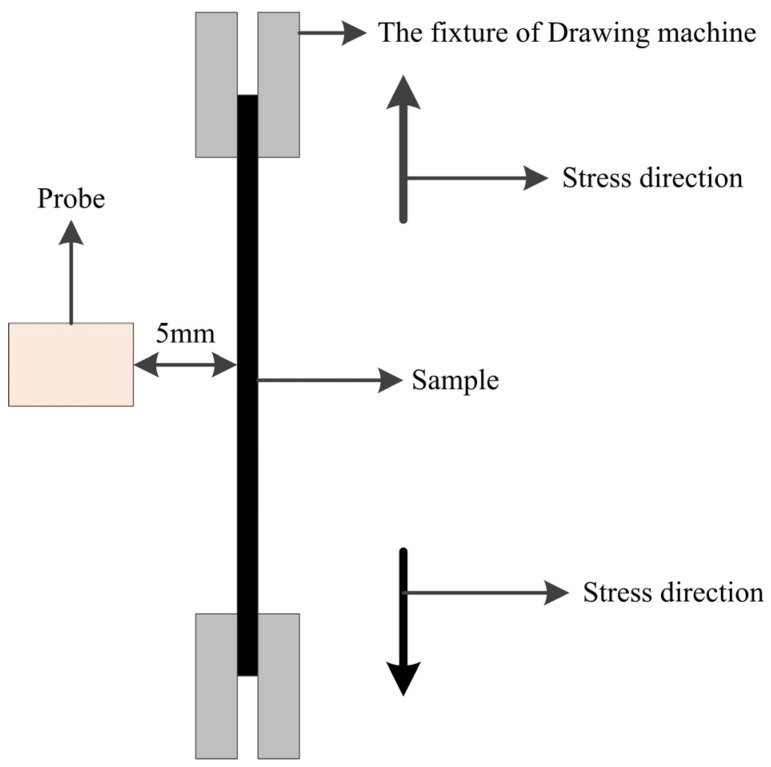
Schematic diagram of testing.

The distance between the 3MA probe and the sample was kept constant at 5 mm, and the magnetic field direction was perpendicular to the stress direction (rolling direction). Then, the samples were subjected to an external stress loading test (Figure 4). Tensile stress is applied in the rolling direction of the samples, and the applied stress is in the range of 5–80 MPa (much less than the yield strength) in order to ensure that no plastic deformation occurs in the sample. The stress gradient was 5 MPa, and each stress state was kept static for 10 min to ensure that both stress and strain were stabilized, and the detection time was not less than 30 s for each stress state.

### 2.3. Metallographic and Magnetic Force Microscope (MFM) Measurements

The microstructure of the alloy can alter the micro-magnetic NDT signal through its effect on domain structure and domain motion. Therefore, metallographic measurements were also performed. The materials were cut into metallographic samples with 10 × 10 mm^2^. Metallographic samples, prepared per the GB/T13298-2015 standard [33], were photographed using an Olympus BX53 optical microscope (Olympus Corporation, Tokyo, Japan).

Atomic force microscopy (AFM) (Benyuan Nano Instrument Co., Ltd., Guangzhou, China) and magnetic force microscope (MFM) (Keens China Limited, Tokyo, Japan) assessments were performed using small specimens (10 × 10 mm^2^) in order to observe the magnetic domains [15,16]. The MFM measurements were performed under ambient conditions using commercial MFM instruments: an MFP-3D atomic force microscope from Asylum Research and a PPP-MFMR-10 probe from Nanosensors. Before the measurements, the samples were polished to achieve a mirror effect and then etched using Nital (96% ethanol + 4% nitric acid) for seven seconds.

The 3D and 2D morphological images show the local height of the sample surface. To study the evolution of the magnetic microstructure of the microstructure and the resulting domain structure, we combined an applied magnetic field with MFM. The magnetic domain images show the local domain orientation and the domain structure of the sample. The direction of the applied magnetic field is parallel to the longitudinal axis of the magnetic domain image. Unfortunately, the evolution of magnetic domains and elastic stresses in this study can only be characterized by micro-magnetic signals because of the difficulty of combining MFM with elastic stresses in ferromagnetic materials.

### 2.4. Pattern Recognition Based on MLP Model

The Multi-layer perceptron (MLP) is a forward-structured artificial neural network that maps a set of input vectors to a set of output vectors. The MLP can be viewed as a directed graph consisting of multiple node layers, each fully connected to the next layer. In addition to the input nodes, each node is a neuron with a nonlinear activation function. A supervised learning approach using BP back propagation algorithm is used to train the MLP, which is a generalization of the perceptron and overcomes the weakness that the perceptron cannot recognize linearly indistinguishable data.

The back propagation-based learning is a typical feed forward network, where the information is processed in a layer-by-layer direction from the input layer to each hidden layer to the output layer. The hidden layer implements a nonlinear mapping to the input space, and the output layer implements a linear classification, and the nonlinear mapping method and linear discriminant function can be learned simultaneously.

**Figure 5:**
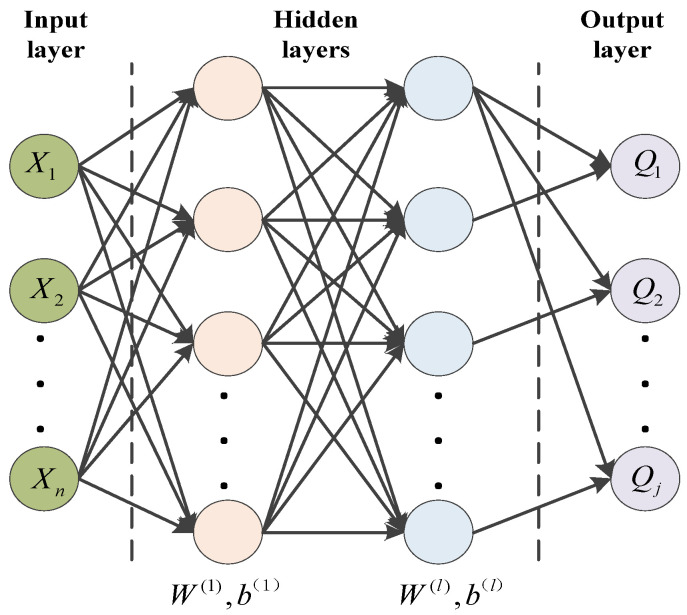
Structure of MLP neural network.

Before the training of MLP neural network, there is no need to clarify the mathematical relationship between the data. Through the input and output of the given training set, the training algorithm will adjust the weight and bias of MLP neural network and update iteratively, so as to reduce the loss function of the model, so as to complete the training. Figure 5 shows the structure of the MLP neural network.

The loss function of MLP can be defined as:

(1) $$L(W)=\underset{x_{i}\in D}{\sum }-y_{i}(w^{T}x_{i}+b);D=\{x_{i}|y_{i}(w^{T}x_{i}+b)<0\}$$

where $y_{i}$ is any piece of data, W is the weight matrix from the l−1st layer to the lth layer, b is the bias matrix from the l−1st layer to the lth layer, and *D* is the training set. The gradient of the loss function can be defined as:

(2) $$\frac{\partial L(W)}{\partial W}=-\underset{x_{i}\in D}{\sum }y_{i}x_{i}$$

Stochastic gradient descent method is used to solve the problem, then the gradient iteration formula can be defined as:

(3) $$W^{\left(t+1\right)}=W^{\left(t\right)}-\alpha_{\phi }y_{i}x_{i}$$

$\alpha_{\phi }$ is learning rate. The MLP model training process can be described as Algorithm 1.
**Algorithm 1** Training steps for the MLP modelInput: Training set $D={\left\{(x^{\left(n\right)},y^{\left(n\right)})\right\}}_{n=1}^{N}$, validation set V, learning rate $\alpha_{\phi }$, number of network layers L, number of neurons $M_{l}$, activation function for each layer $f_{l}$, where 1≤l≤L.1. Randomly initialize the weight matrix W and bias matrix b for each layer;2. Randomly disrupt the samples in the training dataset D;3. For *n* = 1…*N* do;4. Samples $\left(x^{\left(n\right)},y^{\left(n\right)}\right)$ are selected from the training set D as inputs to the MLP network;5. Calculate the input and activation values for each layer;6. Calculate the error for each layer according to the backpropagation algorithm;7. Calculate the gradient for each layer of this sample;8. Update the weight matrix W and bias matrix b for each layer;9. End.10. Until a pre-determined number of iterations is reached, or the model has the required accuracy on the validation set, the model training is completed and the results are output.

## 3. Results and Discussions

### 3.1. Microstructure Features of Samples

**Figure 6:**
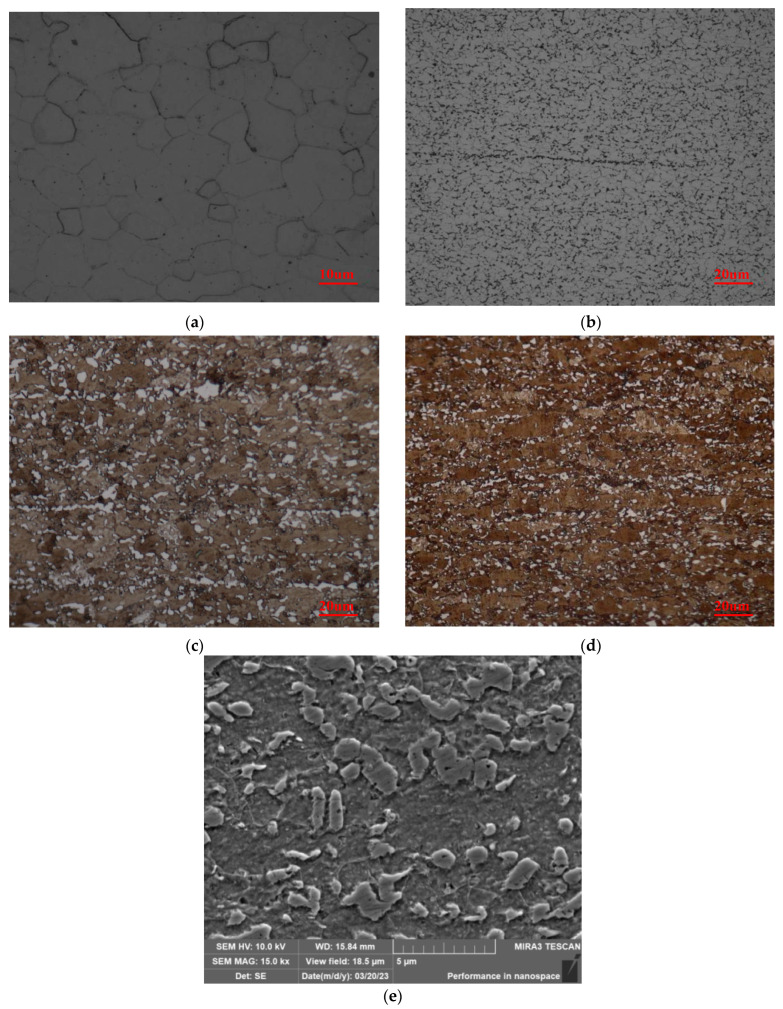
Microstructure of OM samples with different carbon contents. (**a**) Sample 1, (**b**) Sample 2, (**c**) Sample 3, (**d**) Sample 4, and (**e**) SEM imagery of Sample 3.

Figure 6 illustrates the microstructure of the sample (optical metallographic and SEM images). Sample 1 is a low strength steel with a ferrite matrix and also contains a small amount of pearlite and cementite (Figure 6a). Sample 2 is a small-sized granular pearlite (black) and ferrite (white) duplex steel with a small amount of cementite along the crystal (Figure 6b). Samples 3 and 4 are both granular martensite (black) and ferrite (white) duplex steels with significant segregation bands and no obvious grain boundaries in the ferrite (Figure 6c,d).

Sample 1 has a coercivity less than 1000 Oe, which is a soft magnetic material. As the pearlite (martensite) content in the sample increases, the magnetic hardness increases. Therefore, the magnetic properties of samples 2, 3, and 4 are lower than those of sample 1. Based on our previous study [34], the pearlite (martensite) content and pearlite (martensite) grain area in the sample are significant effect on the micro-magnetic NDT signal. And the microstructure characteristics of the samples were obtained using image processing techniques as shown in Table 2 [34].

### 3.2. Characteristics Analysis of MBN and MIP

**Figure 7:**
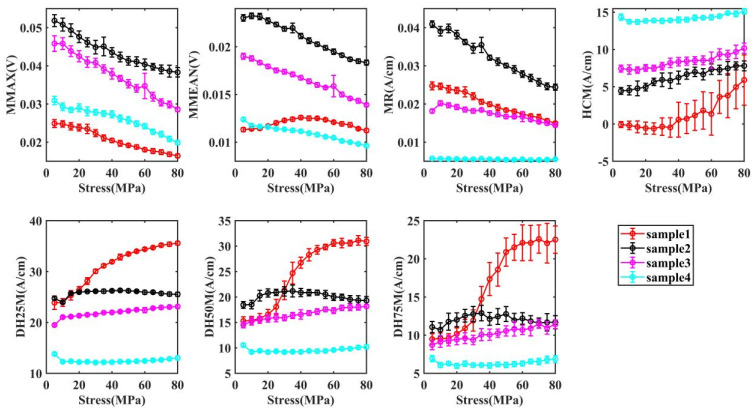
The change of MBN characteristics.

Figure 7 demonstrates the response of MBN characteristics to stress in the elastic strain range, from which it can be concluded that MBN characteristics change monotonically with the increase of stress. Applying tensile stresses in the rolling direction produces the effect of compressive stresses acting in the perpendicular rolling direction. With increasing tensile stress in the rolling direction, the MMAX, the MMEAN, and the MR in the vertical rolling direction in different samples show a monotonically decreasing trend; however, the HCM monotonically increases. The anisotropy of samples increases and the magnetic domains are more likely to move in the direction of the easy magnetization axis (rolling direction), and the external stress further decreases the exchange energy of the magnetic crystals in the rolling direction. In the perpendicular easy magnetization direction, it is more difficult for the domains to move, and the MBN signals are weakened.

**Figure 8:**
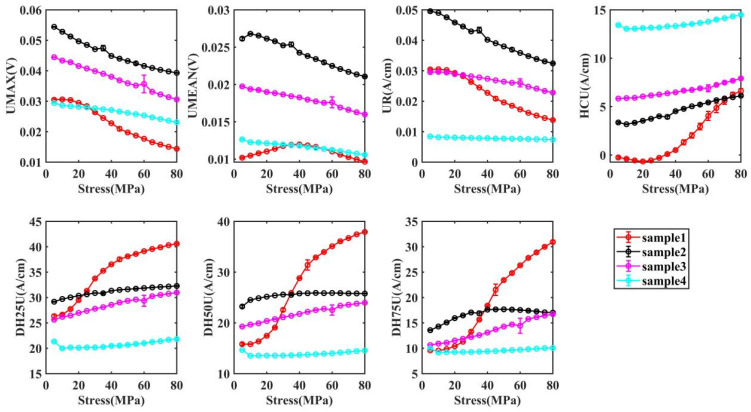
The change of MIP characteristics.

Figure 8 demonstrates the relationship between MIP signal and stress in the elastic strain range for materials with different microstructures, from which it can be concluded that MIP characteristics change monotonically with the increase of stress. Similar to the variation pattern of MBN signal, with the increase of tensile stress in the vertical rolling direction, the UMAX, the UMMEAN and the UR in the rolling direction show a monotonically decreasing trend; however, the HCU monotonically increases.

The different microstructures have different lattice interaction forces, resulting in different material response to stress, and the resulting magnetic domain structure and domain motion properties are different, resulting in differences in magnetic properties [34].

With both the reversible and irreversible motion of magnetic domains, the variability of sample 1 with external stress is greater compared to that of samples 2, 3, and 4. In terms of the slope of coercivity and width of butterfly curve change, sample 1 shows a significantly higher growth rate after 35 MPa than before 35 MPa.

For sample 1, in the elastic range, the micro-magnetic NDT signal can be divided into two stages. The microscopic residual stress interacts with the external stress and the internal stress has a greater effect on the magnetic domain motion than the external stress. The compressive stress applied in the magnetic field direction increases the domain wall exchange energy in the magnetic field direction, but does not change the domain structure, and it is more difficult for the domain to move in the magnetic field direction at the same magnetization strength, thus the peak value is reduced and the coercivity field is increased. However, when the external stress exceeds the internal stress limit, the external stress causes the ferrite to undergo elastic deformation (rolling direction). The ferrite elastic deformation leads to a longer path of domain motion in the rolling direction and a shorter path of domain motion in the magnetic field direction, which further increases the domain wall exchange energy and prevents the short path of domain motion from generating higher and more concentrated MBN pulses, resulting in a lower MMAX, higher HCM, and increased width of butterfly curve. However, unlike the irreversible motion, the reversible motion of the magnetic domains is less driven and therefore more sensitive to the resistance [35]. In sample 1, the inflection point appears around 25 MPa, while in MBN, it is 35 MPa [35].

Samples 2–4 show an increase in the content of ferrite (or martensite) and a decrease in grain size, which leads to a significant increase in dislocation density. At this point, external stresses (5–80 MPa) are not able to deform the microstructure. Therefore, samples 2–4 do not show the trend of sample 1.

### 3.3. Results of Magnetic Domain Observations

**Figure 9:**
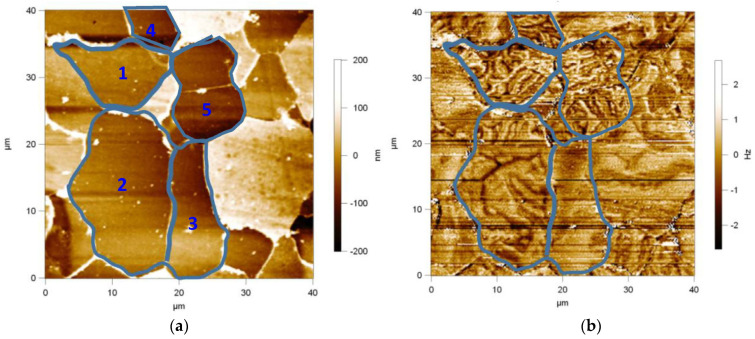
Magnetic domain morphology of sample 1. (**a**) 2D Topography, (**b**) Magnetic domain morphology.

For sample 1, the height difference exists on the sample surface because the ferrite is easily corroded after corrosion and the carbon-rich phase (cementite Fe3C, pearlite, etc.) is not easily corroded (Figure 9a). At the same time, the presence of carbon-rich phase makes the lattice defective, and dislocation density increases while lattice distortion energy increases, thus increasing the microscopic residual stress of the material. During the AFM inspection, the MFM probe encounters higher tissue and produces the black lines in the figure (Figure 9b).

The domains in Figure 9b are mainly ferrite domains (lamellar (3), dendritic (2), labyrinthine (1, 4 and 5)), while a few cementite domains surrounding the ferrite grain boundaries appear as small black beads. In Figure 9b, it is concluded that the magnetic domains in all grains, except for grain 3, show the phenomenon of domain separation. A large amount of carburite is dispersed in grain 2, which leads to the phenomenon of domain separation. The magnetic lines of the carbon-rich phase (cementite) within the crystal are not closed [15,16], forming unclosed magnetic domains, which increases the demagnetization energy. To reduce the demagnetization energy, the ferrite domains are subdivided to increase the number of domains, which may be accompanied by the appearance of supplementary domains in the process to close the magnetic lines of force. Grains 1, 4, and 5 show the phenomenon of domain separation, which may be related to the crystal orientation. For ferrite (three easy magnetization axes), the direction of [100] is the easiest magnetization axis and requires the lowest energy. Therefore, there is generally no magnetic domain separation in the [100] direction. In contrast, the crystals are in the direction of the other easy magnetization axes, which require higher energy. The energy of the demagnetizing field is reduced by the magnetic domain subdomain.

Applying tensile stress in the direction of the easy magnetization axis changes the microscopic residual state (no elastic deformation of the microstructure) and reduces the exchange energy of the easy magnetization axis, causing the MBN and MIP signals in the perpendicular easy magnetization axis direction to decrease. After a certain threshold is exceeded, the ferrite elongates in the direction of the easy magnetization axis, which decreases the magnetic domain motion path in the vertical direction (at this point, the external stress has a greater effect on the magnetic domain motion than the microstructure). Therefore, the MBN and MIP signals weaken. Stresses exceeding the elastic limit cause plastic deformation of the ferrite, which causes lattice rupture and a further increase of dislocation density [36], and MBN and MIP are weakened again. However, there is a difference in the physical principles of these two cases.

**Figure 10:**
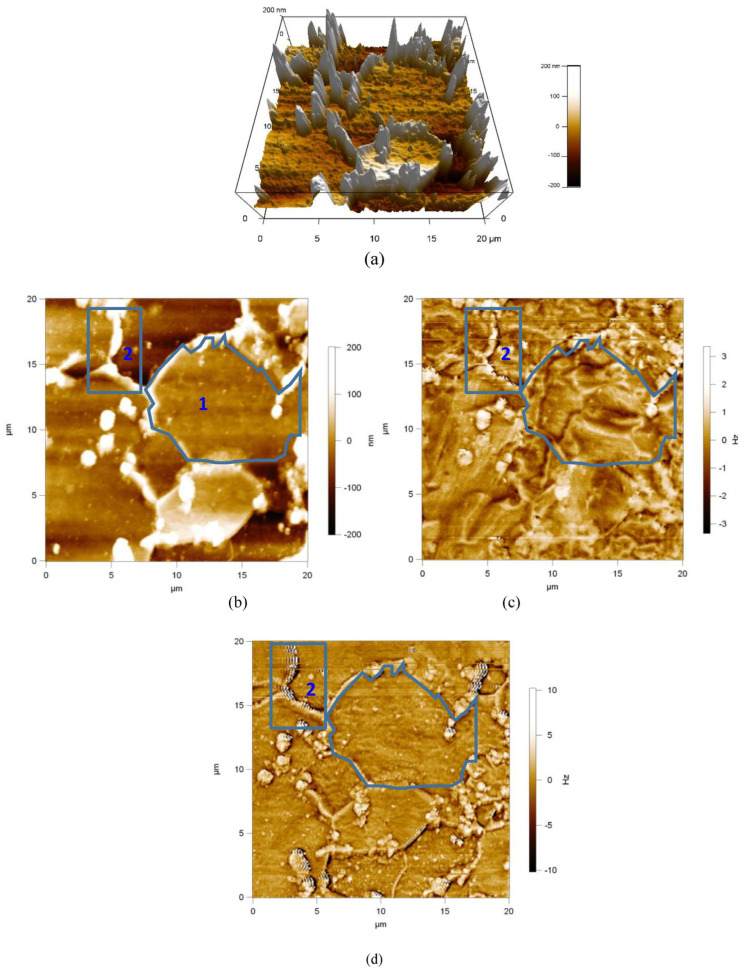
Magnetic domain morphology of sample 2. (**a**) 3D Topography, (**b**) 2D Topography, (**c**) Magnetic domain morphology of 0 Oe, and (**d**) Magnetic domain morphology of 200 Oe.

Figure 10 shows the domain structure of sample 2. For sample 2, granular pearlite and ferrite dominate, and the structure of the granular pearlite magnetic domains can be clearly seen in the figure. Before magnetization, it can be noticed that the domains are divided in grain 1 (ferrite organization) and that multiple granular pearlite magnetic domains in region 2 (white area, Figure 10b) are black and white. After applying a magnetic field of 200 Oe, the magnetic domains in grain 1 are deflected to the same direction, and multiple granular pearlite magnetic domains in region 2 appear to have a laminar structure. It can be concluded that the ferrite domains in the granular pearlite are deflected, and the motion is different from that of the domains in grain 1 due to the presence of the cementite domains, implying that the magnetic domains within the pearlite require higher energy for their motion than the ferrite domains.

The crystal structure affects not only the magnetic domain structure and motion properties, but also the mechanical properties. Compared with ferrite, granular pearlite has a different crystal structure and is harder, less plastic, and more difficult to deform [34]. Under stresses of 5–80 MPa, ferrite in sample 2 was not deformed, so it showed more consistent variation.

**Figure 11:**
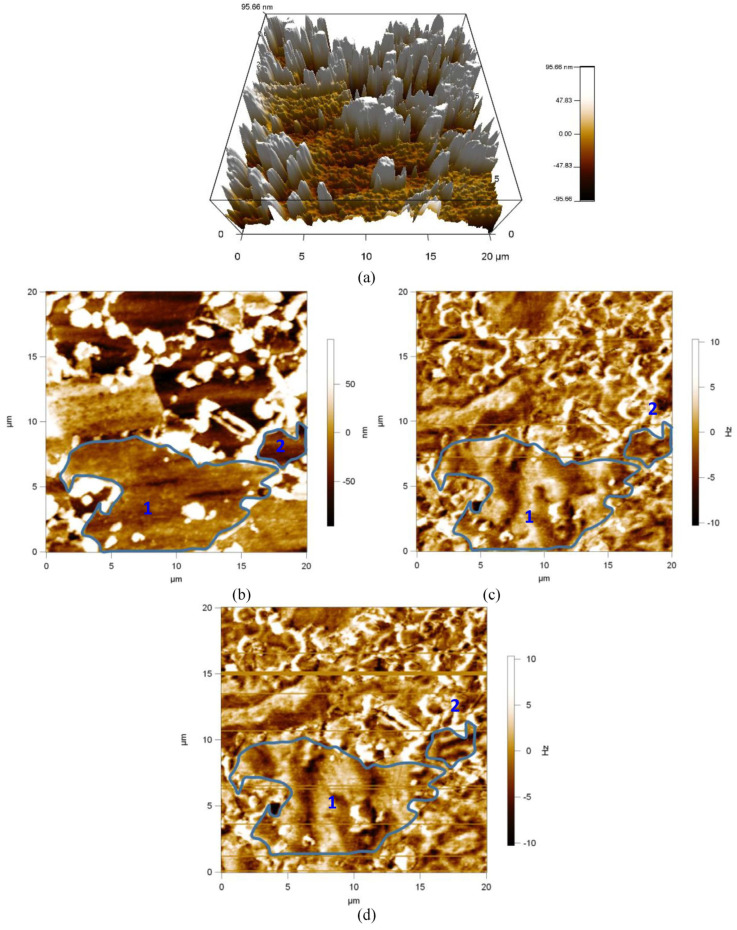
Magnetic domain morphology of sample 3. (**a**) 3D Topography, (**b**) 2D Topography, (**c**) Magnetic domain morphology of 0 Oe, and (**d**) Magnetic domain morphology of 200 Oe.

**Figure 12:**
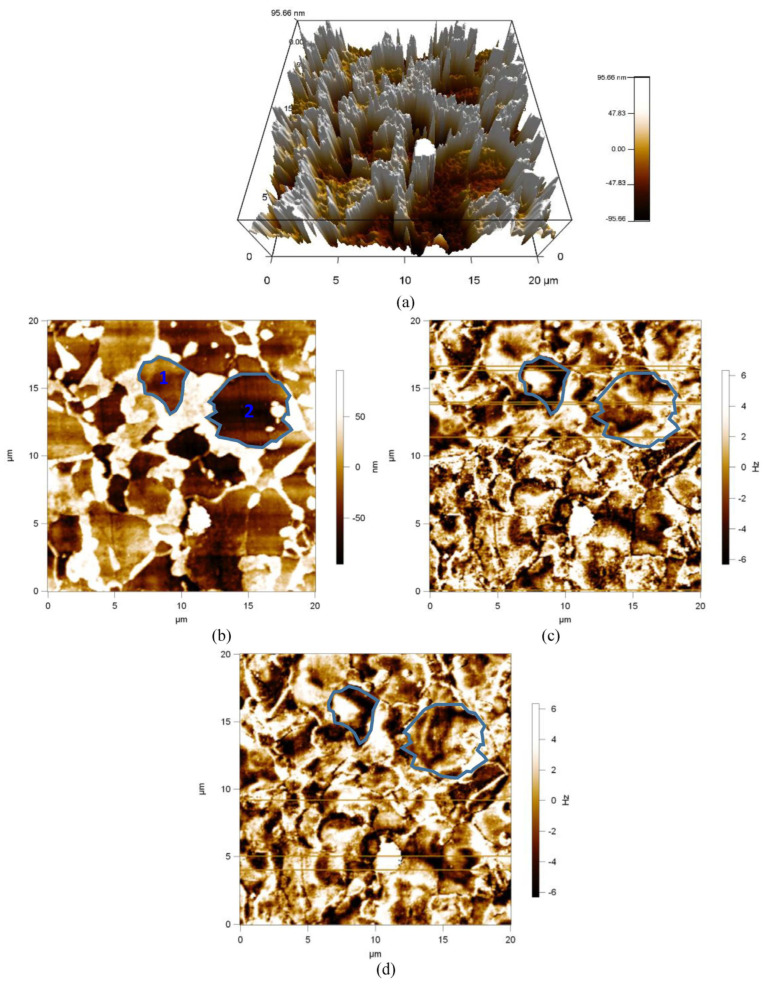
Magnetic domain morphology of sample 4. (**a**) 3D Topography, (**b**) 2D Topography, (**c**) Magnetic domain morphology of 0 Oe, and (**d**) Magnetic domain morphology of 200 Oe.

The microstructure of samples 3 and 4 is dominated by ferrite and martensite (Figure 11 and Figure 12). The martensitic magnetic domains (Figure 11b and Figure 12b, white area) hardly move at a magnetic field strength of 200 Oe (Figure 11d and Figure 12d). Essentially, martensite is also a mixture of cementite and pearlite chemicals, but is more complex than pearlite and with a higher dislocation density. The martensite domain is also an unclosed domain similar to the cementite domain, and in samples 3 and 4, the ferrite domain is more affected by martensite due to the absence of grain boundaries. White areas similar to martensitic domains appear in the ferrite domains (1 and 2, Figure 12) and the morphology of the domains does not change at a magnetization intensity of 200 Oe. As shown in Figure 11b, 1 and 2, the ferrite domains both show domain splitting. At a magnetization intensity of 200 Oe, the magnetic domains in grain 2 in Figure 11b show stripes, which are called striped magnetic domains. It shows that the ferrite magnetic domains are more difficult to move under the same magnetic field excitation in the duplex steel with ferrite and martensite than in the duplex steel with pearlite and ferrite (Figure 10d, Figure 11d and Figure 12d).

Compared with pearlite, martensite is characterized by higher hardness (374 HV) dislocation density, but lower toughness and less deformation. Under the stress condition of 0~80 MPa, the microscopic residual stress equilibrium state of samples 3 and 4 is less affected, i.e., the deformation is smaller in the perpendicular rolling direction, and the magnetic domain motion path changes less, so the magnetic domains are less affected by the compressive stress under the excitation of the external magnetic field; therefore, the micro-magnetic characteristics, such as UR and HCU, are basically stabilized in a horizontal line. The MBN and MIP signals of samples 3 and 4 are lower than those of sample 2, indicating that there is an inflection point in the influence of microstructure on the reversible and irreversible motions of magnetic domains. The martensitic dislocation density is greater than that of pearlite, and the pearlite dislocation density is greater than that of ferrite. Within the sample, the impedance of the reversible and irreversible motions of the magnetic domains increases sequentially, and the MBN and MIP signals weaken above a certain threshold.

**Figure 13:**
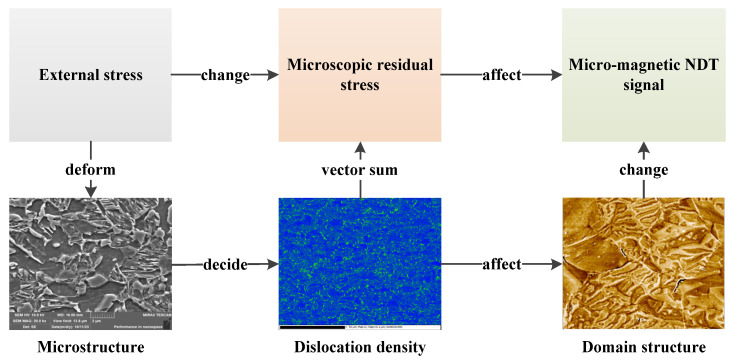
Mechanisms of stress-altered micro-magnetic signals.

The mechanism by which the micro-magnetic signal is altered by stress is shown in Figure 13. The external stresses change the micro-magnetic signal by affecting the deformation of the microstructure, which, in turn, affects the magnetic domain structure or the path of motion. Dislocations are a manifestation of microscopic stresses (caused by crystal defects) in materials, and their vector sum with crystal deformation, etc., is the microscopic residual stress.

In the study, at low stress levels, the external stress first changes the dislocation equilibrium state and thus affects the microscopic residual stress (MBN and MIP signals change in sample 1). when the external stress increases to a certain degree, the external stress changes the crystal shape, causing the crystal to undergo elastic deformation, and then the magnetic domain motion path changes, resulting in the micro-magnetic signals changing accordingly. When the external stress continues to increase, the crystal undergoes plastic deformation, which leads to lattice breakage (increased defects) and thus increased dislocation density (increased microscopic residual stress) [36]. Meanwhile, the magnetic domain structure, motion path, and motion characteristics are changed, and the micro-magnetic signal is also changed.

In different microstructures, the lattice interaction forces and dislocation densities are different even without the influence of external stresses. Therefore, the effects of different microstructures on the structure and motion characteristics of magnetic domains are also different, while the micro-magnetic signals are also different. Due to the different lattice interaction forces and dislocation densities in different microstructures, the microscopic residual stress equilibrium state and the organizational deformation state are different under the same stress. As a result, the trends of the micro-magnetic signals are also different.

### 3.4. Definition and Quantification of Magnetic Domain Characteristics

The MBN and MIP signals mainly originate from the motion of ferrite magnetic domains, while the magnetic domains of other phases directly affect the structure and motion properties of the ferrite magnetic domains. The magnetic domains of other phases (cementite domains in sample 1, pearlite domains in sample 2, and martensite domains in samples 3 and 4) in the MFM image at zero magnetic field strength are identified as magnetic domain features using image processing techniques. This not only reflects the influence of crystal structure and dislocation density on magnetic domains, but also reflects the influence of external stress on the structure and motion characteristics of magnetic domains to some extent.

The phase of cementite in sample 1, pearlite in sample 2, and martensite in samples 3 and 4 is special compared to that of ferrite, and the dislocation density is higher than that of ferrite, both of which results in a higher demagnetization energy than that of ferrite. Magnetic domains in ferrite crystals are split into small-sized domains (domain division phenomenon), which surround the other phase domains to close the unclosed magnetic lines of force. Thus, the magnetic domains are subdivided to reduce the energy of the demagnetizing field. Meanwhile, the magnetic domains of these tissues are not only difficult to be magnetized, but also affect the motion of the surrounding ferrite magnetic domains. Magnetic domains are characterized as the percentage of domain area that is difficult to magnetize in an external magnetic field of less than 200 Oe. In addition, the magnetic domain characteristics defined in this investigation not only characterize the effect of dislocation density on the magnetic domains, but also provide feedback on the effect of external stress on the magnetic domains.

**Figure 14:**
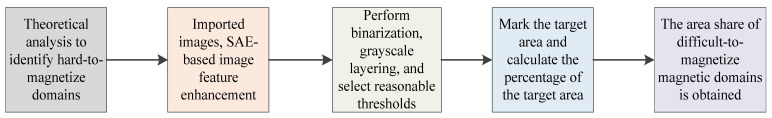
Architecture of magnetic domain feature extraction.

Image gray level layering technique based on Python was used for magnetic domain feature extraction [34]. First, the image is binarized by choosing a suitable threshold value, and then the area share of the target pixels is extracted as the area share of the difficult-to-magnetize magnetic domains (Figure 14) [34] The quantitative results are shown in Table 3.

**Figure 15:**
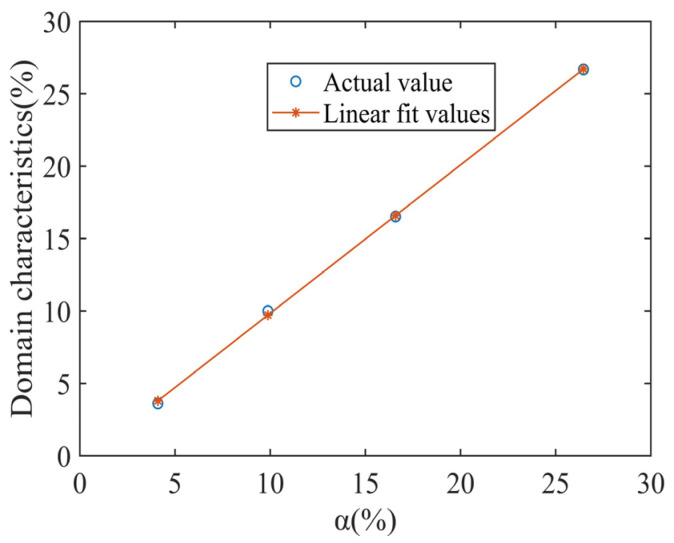
Correlation of feature microstructure with magnetic domain characteristics.

By comparing Table 2 and Table 3, it can be seen that the characteristic microstructure (α) has a strong consistency with the magnetic domain characteristics (Figure 15), and the relationship is almost linear. The fitting relationship is as follows: *y* = 1.2048 *x −* 0.4066.

As can be seen from Table 4, the largest error in the three sets of validation data is 0.26%, and the smallest error is 0.01%. It is concluded that this fitting relationship can more accurately characterize the microstructure characteristics and magnetic domain characteristics of the trend.

### 3.5. Pattern Recognition Results

In the process of data processing, it is found that under 5 MPa stress gradients, the accuracy of pattern recognition results (the degree of agreement between the predicted label and the actual label) is only 12.7%. Therefore, the micro-magnetic characteristic data at stresses such as 5 MPa, 15 MPa, …, 75 MPa, etc. are removed from the sample set to extend the stress gradient. Finally, the filtered sample set (micro-magnetic data and corresponding microstructure and stress data) has a total of 7700 pieces.

According to the previous section, microstructure (pearlite content, martensite content, grain size, etc.) and external stresses have a direct influence on micro-magnetic NDT. A set of microstructures and stress states has a set of corresponding micro-magnetic characteristics. In theory, the corresponding microstructure features and stress states can be quickly determined based on the micro-magnetic features. On the one hand, the microstructure features and stresses are coupled, and on the other hand, there are countless combinations of microstructure and stress states. In order to describe the microstructure and stress state in a simple and uniform way, the set of microstructure features and stress states is defined as a pattern (a label), which is the patterned representation of microstructure features and stress states. Then, the dataset is composed of micro-magnetic features and labels. According to different microstructures and stress gradients, the samples were divided into 32 categories (Table 5).

The two-hidden-layer (25, 25) MLP model was adopted. The hidden layer function is ‘tansig’, the output layer function is ‘purelin’, and the network training function is ‘trainlm’. Based on experience, the maximum number of iterations of the MLP model is 1000. The learning efficiency is 0.1 and the target error is 0.00001.

The sample set is divided into a training set and a test set, and the number of training set samples is 5200. The test set is prepared using sample data of the same steel grade as sample 1 and sample 3 but belonging to different batches. The number of samples is 2500.

**Figure 16:**
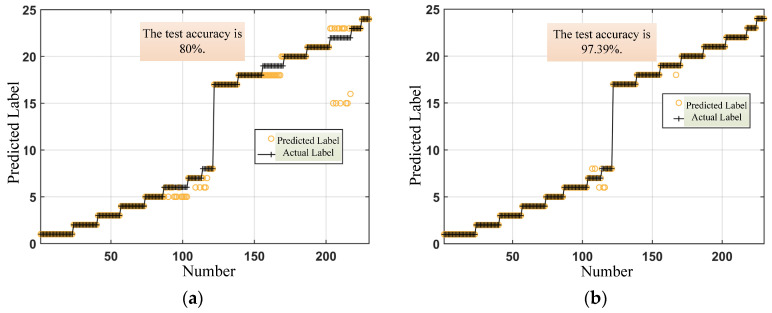
Pattern recognition results. (**a**) Pattern recognition results without magnetic domain features; (**b**) Pattern recognition results with magnetic domain features added.

As shown in Figure 16a, the prediction accuracy of the MLP model without adding magnetic domain features is 80%. By taking the magnetic domain features together with the micro-magnetic features as input for pattern recognition, the prediction accuracy of the MLP model is 97.39% (Figure 16b), which is 17.39% higher than that of micro-magnetic features alone.

In addition, as shown in Figure 16a, part of the pattern recognition result is label 15, while the actual label is 23. After querying the original data, it is found that the micro-magnetic features corresponding to the actual labels of 15 and 23 are cross-interfering, which leads to the wrong result of pattern recognition. However, the labels 15 and 23 can be distinguished, after adding the magnetic domain feature.

The experimental results show that the pattern recognition accuracy of the magnetic domain feature and the micro-magnetic feature together as input is higher than that of the micro-magnetic feature as the input alone. The pattern recognition method proposed in this investigation takes the effects of microstructure and stress on micro-magnetic signals into account, which realizes the quantitative characterization of microstructure features and stress states.

## 4. Conclusions

In this study, the coupling of characteristic microstructures and stresses and the pattern recognition of microstructures characteristic and stresses are investigated using MIP and MBN techniques. The following conclusions are drawn.

Firstly, the response of different microstructures to stress, and the effects of microstructures and stress on magnetic domains and micro-magnetic features, is investigated. The experimental results show that applying tensile stress perpendicular to the magnetic field direction increases the resistance of the magnetic domains to the magnetization direction, which reduces the magnetism in the magnetization direction and leads to the weakening of the micro-magnetic signals. The results also show that different crystal structures lead to different interaction forces between the crystals and the morphology of the magnetic domains, which results in different trends of micro-magnetic features under the influence of stress.

Secondly, the quantitative characterization of microstructure features and magnetic domain characteristics is realized. The magnetic domain features are defined based on the magnetic domain structure and motion, and the image processing technique is used to extract the magnetic domain features. Then the relationship between the microstructure features and the magnetic domain features is established and verified. The results show that the magnetic domain features have higher sensitivity than the multi-source micro-magnetic features.

Finally, the pattern recognition of microstructure and stress is realized based on magnetic domain features and micro-magnetic features. The pattern characterization method of microstructure features and stress is proposed, and the MLP model is introduced to realize the pattern recognition of microstructure features and stress based on magnetic domain features and micro-magnetic features (the testing accuracy being more than 97%). The results show that the pattern recognition accuracy of magnetic domain features and micro-magnetic features as input parameters is higher than that of micro-magnetic features alone.

## 5. Declaration of Competing Interest

We declare that we have no financial and personal relationships with other people or organizations that can inappropriately influence our work; there is no professional or other personal interest of any nature or kind in any product, service, and/or company that could be construed as influencing the position presented in, or the review of, the manuscript entitled ‘‘Stress and microstructures characterization based on Magnetic incremental permeability and magnetic Barkhausen noise techniques”.

## Figures and Tables

**Table 1:** Material formulation and mechanical properties.

Sample Number	Rp0.2 (MPa)	Rm (MPa)	A (%)
1	163.00	286.00	46.40
2	458.00	578.00	25.20
3	397.00	645.00	26.20
4	466.00	838.00	15.80

Note: yield strength (Rp0.2), tensile strength (Rm) and elongation at break (A).

**Table 2:** Quantitative results of microstructure features.

Sample Number	Ferrite grain Boundary	Average Grain Area (${\mu m}^{2}$)	Percentage of Pearlite or Martensite (%)
1 (pearlite)	1	6.57	4.1
2 (pearlite)	1	3.97	9.88
3 (martensite)	2	5.29	16.59
4 (martensite)	2	5.06	26.46

Note: The percentage of pearlite or martensite is defined as *α*; Average grain area of pearlite or martensite is defined as *β*.

**Table 3:** Quantitative results of magnetic domain characteristics.

Samples	Microstructures	Magnetic Domains	Domains Characteristics
1	Ferrite; pearlite	Closed domains; unclosed domains	3.62%
2	Ferrite; granular pearlite	Closed domains; closed domains and unclosed domains	10%
3	Ferrite, martensite	Closed domains; unclosed domains	16.52%
4	Ferrite, martensite	Closed domains; unclosed domains	26.68%

**Table 4:** Magnetic domain characterization validation results.

Samples Number	Real Value	Projected Value	Error
1	30.2%	29.94%	0.26%
2	3.3%	3.35%	−0.05%
3	1.60%	1.59%	0.01%

**Table 5:** Modelled quantitative results.

Samples Number	Ferrite Grain Boundary	*α* (%)	*β* (${\mu m}^{2}$)	Stress (MPa)	Label
1	1	4.1	281	10	1
1	1	4.1	281	20	2
……		……	……	……	……
1	1	4.1	281	80	8
……		……	……	……	……
4	2	26.46	54.32	80	32

## Data Availability

The raw data supporting the conclusions of this article will be made available by the authors on request.
